# Discriminatory Attitudes and Practices by Health Workers toward Patients with HIV/AIDS in Nigeria

**DOI:** 10.1371/journal.pmed.0020246

**Published:** 2005-07-19

**Authors:** Chen Reis, Michele Heisler, Lynn L Amowitz, R. Scott Moreland, Jerome O Mafeni, Chukwuemeka Anyamele, Vincent Iacopino

**Affiliations:** **1**Physicians for Human RightsBoston, MassachusettsUnited States of America; **2**Department of Internal Medicine, School of Medicine, and Ann Arbor Veterans' Affairs Health System, University of MichiganAnn Arbor, MichiganUnited States of America; **3**Brigham and Women's Hospital and Harvard Medical SchoolBoston, MassachusettsUnited States of America; **4**Policy ProjectMaitamaNigeria; **5**Center for the Right to HealthShomoluNigeria; University of Cape TownSouth Africa

## Abstract

**Background:**

Nigeria has an estimated 3.6 million people with HIV/AIDS and is home to one out of every 11 people with HIV/AIDS worldwide. This study is the first population-based assessment of discrimination against people living with HIV/AIDS in the health sector of a country. The purpose of this study was to characterize the nature and extent of discriminatory practices and attitudes in the health sector and indicate possible contributing factors and intervention strategies. The study involved a cross-sectional survey of 1,021 Nigerian health-care professionals (including 324 physicians, 541 nurses, and 133 midwives identified by profession) in 111 health-care facilities in four Nigerian states.

**Methods and Findings:**

Fifty-four percent of the health-care professionals (550/1,021) were sampled from public tertiary care facilities. Nine percent of professionals reported refusing to care for an HIV/AIDS patient, and 9% indicated that they had refused an HIV/AIDS patient admission to a hospital. Fifty-nine percent agreed that people with HIV/AIDS should be on a separate ward, and 40% believed a person's HIV status could be determined by his or her appearance. Ninety-one percent agreed that staff and health-care professionals should be informed when a patient is HIV-positive so they can protect themselves. Forty percent believed that health-care professionals with HIV/AIDS should not be allowed to work in any area of health-care that requires patient contact. Twenty percent agreed that many with HIV/AIDS behaved immorally and deserve the disease. Basic materials needed for treatment and prevention of HIV were not adequately available. Twelve percent agreed that treatment of opportunistic infections in HIV/AIDS patients wastes resources, and 8% indicated that treating someone with HIV/AIDS is a waste of precious resources. Providers who reported working in facilities that did not always practice universal precautions were more likely to favor restrictive policies toward people with HIV/AIDS. Providers who reported less adequate training in HIV treatment and ethics were also more likely to report negative attitudes toward patients with HIV/AIDS. There was no consistent pattern of differences in negative attitudes and practices across the different health specialties surveyed.

**Conclusion:**

While most health-care professionals surveyed reported being in compliance with their ethical obligations despite the lack of resources**,** discriminatory behavior and attitudes toward patients with HIV/AIDS exist among a significant proportion of health-care professionals in the surveyed states. Inadequate education about HIV/AIDS and a lack of protective and treatment materials appear to contribute to these practices and attitudes.

## Abstract 2

Results of a survey of attitudes and actions of over 1000 Nigerian health care professionals (doctors, nurses, and midwives).

## Introduction

With an estimated 3.5 million people with HIV/AIDS, Nigeria is home to one of every 11 of the 40 million people with HIV/AIDS worldwide [1]. The HIV prevalence among adults in Nigeria has increased from 1.8% in 1991 to an estimated 5.8% in 2001 [1]. Prevalence ranges from 2% to 14.9% in the country's 36 states and Federal Capital Territory [2]. According to official estimates, Nigeria has an estimated 3.6 million people with HIV/AIDS and approximately 310,000 AIDS deaths this year alone [3], and these numbers are projected to increase each year. In 1999, with the election of President Olusegun Obasanjo, Nigeria emerged from approximately 20 years of military dictatorship in which little governmental attention or funding was directed at addressing HIV/AIDS [4].

People living with HIV/AIDS (PLWA) in Nigeria have been found to be subject to discrimination and stigmatization in the work place, and by family and communities [5,6]. PLWA may also face discrimination from those employed in the health-care sector. [5]. Discriminatory or unethical behavior by health-care professionals against PLWA, as documented in other countries [7–11], may create an atmosphere that interferes with effective prevention and treatment by discouraging individuals from being tested or seeking information on how to protect themselves and others from HIV/AIDS [12–14]. Furthermore, discriminatory practices and violations of international principles of medical ethics may serve to legitimize other forms of discrimination against people living with HIV/AIDS.

Anecdotal information suggests that health-care professionals in Nigeria may engage in discrimination against and stigmatization of PLWA [6,15]. The prevalence, character of, and factors contributing to these practices are, however, largely undocumented. To address this, Physicians for Human Rights (PHR), Policy Project Nigeria, and the Center for the Right to Health conducted a survey of health professionals in four sites in Nigeria. The study was designed to answer three research questions. (1) Are there discriminatory practices in the health sector that affect the health and well-being of people with HIV/AIDS in Nigeria? (2) How receptive are health workers and institutions to treating people with HIV/AIDS? And (3) what underlying factors may contribute to any discriminatory practices? The study was intended to inform ongoing policy discussions and development of effective interventions.

## Methods

### Sampling

At the time of the study, approximately 120,000,000 people were living in the 36 states and Federal Capital Territory of Nigeria [16]. We conducted the study in four states: Abia, Gombe, Kano, and Oyo. These sites were selected by dividing the country's six geopolitical zones into two sections—north and south—in order to capture geographical and other differences and then randomly selecting two of three zones from each section. Within the four selected zones, using health-care facility lists compiled by Nigeria's Federal Ministry of Health [17], we identified states that have a tertiary care institution and randomly selected one of these states from each zone. To obtain a representative sample of health-care professionals, we proportionally sampled doctors, nurses, and midwives from the tertiary facility and systematically selected public and private secondary and primary health-care facilities in the four states. Fifty-four percent of the health-care professionals were sampled from tertiary care facilities. We determined the sample size based on local activists' estimates that 10% of clinicians have discriminatory behavior and attitudes, a margin of error of ± 0.01% and a 90% confidence (10% significance) level. The sample size required given these constraints was 301 health-care professionals. However, our sample design included several levels of clustering, and we therefore assumed a design effect of three and thus the sample size needed was calculated to be approximately 1,000. Eligible facilities were medical facilities included in the published federal government database, which indicated that there were 2,585 health facilities in the four states [17]. In each health-care facility, we systematically sampled from all doctors, nurses, and midwives to acquire information about their knowledge, attitudes, and behavior. Eligible professionals were physicians or certified nurses or midwives working in positions with direct patient contact. Data on the number of health-care professionals were derived from Federal Ministry of Health data, which indicated that these four states have a total of nearly 4,500 health-care professionals who serve a population of approximately 17.8 million people [18].

### Survey Questionnaire

The 104-item health-care professional survey included questions on respondent demographics; practices regarding informed consent, testing, and disclosure; treatment and care of patients with HIV/AIDS; and attitudes and beliefs about treatment and care of patients with HIV/AIDS including informed consent, testing, and disclosure.

Treatment and care practices of patients with HIV/AIDS were assessed using Likert-type scales (e.g., the possible answers were “always,” “most of the time,” “sometimes,” “rarely,” and “never”). Attitudes and beliefs were assessed by a response of “agree” or “disagree” with statements regarding testing, treatment, and care of patients with HIV/AIDS.

Using a separate 103-item survey instrument, we obtained information about each facility's capacity, resources, and policies from the person in charge of the facility. The questionnaires were written and interviews conducted in English. Seven regional, human rights, and medical experts reviewed the questionnaires for content validity. The instruments were pilot tested among 20 participants in Lagos and suggestions regarding clarity and cultural appropriateness were incorporated.

### Interviewers

After completing an intensive training program, 24 Nigerian surveyors conducted the survey interviews. Interviewer training consisted of 5 d of classroom teaching and role-playing followed by several days of field observation and ongoing supervision by PHR and Nigerian researchers.

All interviews were conducted over 5 wk in October and November 2002. Interviews lasted approximately 20–30 min and were conducted in the most private setting possible within each health-care facility. All questionnaires were reviewed for completeness and for correctness of recording after the interview by the interviewers themselves, by the Nigerian research team leaders, and by PHR field supervisors at the end of each day.

### Definitions

In the surveys, informed consent was defined as ensuring that a patient who is competent to make decisions is informed and consulted about his or her care. Respondents were informed that this included the responsibility of the clinician to let the patient know about any procedure or medical decision, reasonable alternatives to it, and the risks, benefits, uncertainties, and possible consequences related to each alternative. The clinician must carry out the discussion in layperson's terms, assess the patient's understanding along the way, and ensure that the patient understands the information and consents to it voluntarily [19]. Universal precautions were defined as the use of protective barriers such as gloves, gowns, aprons, masks, or protective eyewear, which can reduce the risk of exposure to potentially infective materials at all times regardless of a patient's HIV or other status [20].

### Human Subjects Protection

This study was reviewed and approved by an independent ethics review board of individuals with expertise in clinical medicine, public health, bioethics, and international HIV/AIDS and human rights research developed for this research project by PHR. In reviewing the research, the review board was guided by the relevant provisions of Title 45 of the US Code of Federal Regulations [21], and complied with the Declaration of Helsinki, as revised in 2000 [22]. The study was also reviewed for ethical and cultural appropriateness by a panel convened in Nigeria by Policy Project Nigeria. In addition, permission for the study and access to facilities was granted by the Nigerian Federal Ministry of Health, state and local government authorities, and facility directors. There were no limitations placed on movement or surveying. Verbal informed consent was obtained from all participants, their names were not recorded, and only minimal identifying information was taken in order to preserve the anonymity of their responses. Participants did not receive any compensation.

### Statistical Analysis

The data were analyzed using Stata 7 [23]. To control for clustering and design effect, the sample was weighted by the number of states selected with a tertiary facility from each of six selected geopolitical zones, the number of local government areas per location, the number of facilities selected from each local government area, and the response rate in each location. The study's principal objective was to describe health-care professional practices and attitudes towards people with HIV/AIDS, rather than to conduct comparisons between professionals or explore associations between professional characteristics and different outcomes. However, we conducted bivariate analyses using chi-square analyses and simple logistic regression to compare negative practices and attitudes among the three health specialties surveyed (doctors, nurses, and midwives) and to test for associations between reported facility resources and providers' reported adequacy of AIDS training and reported negative practices and attitudes about HIV/AIDS.

## Results

### Characteristics of Facilities

Of the 163 facilities sampled, 20 were no longer operational; for ten, contact could not be established after two attempts at the time of sampling; and 15 were not eligible. Of the 118 eligible facilities where contact was established, 111 participated in the study (78% of operational facilities). Over half of the facilities were general hospitals (54%) and 23% were primary health centers.

Eighty-four percent of facility directors reported not having antiretroviral medications in their facility. Moreover, the availability of other medications and dietary supplements was limited, and protective materials and other supplies and utilities were not always available (Table 1).

**Table 1:**
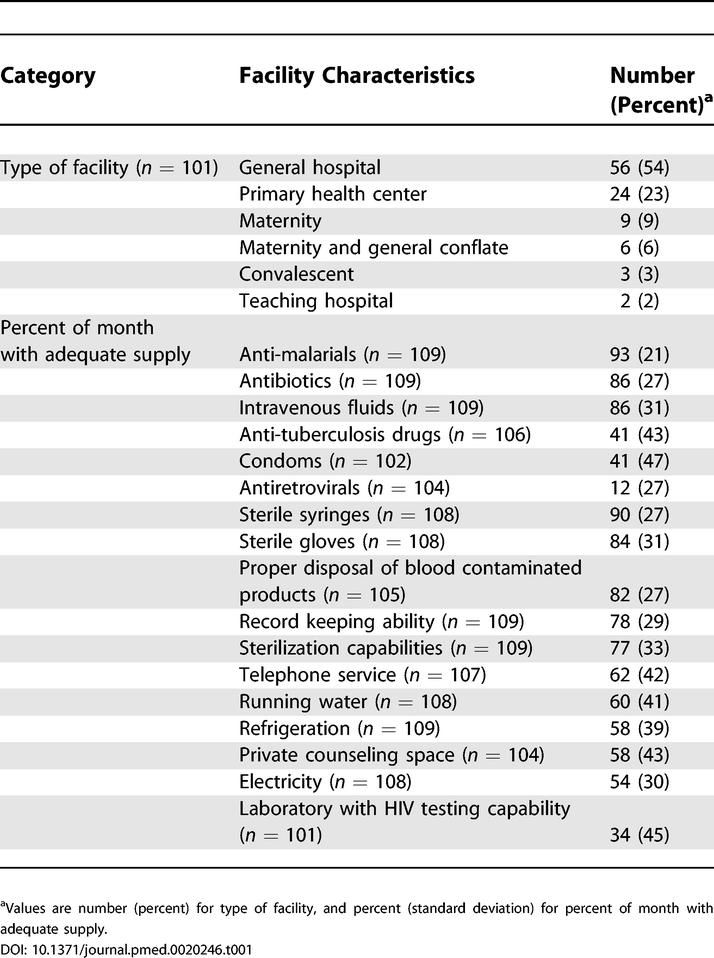
Characteristics of 111 Participating Facilities

### Characteristics of Respondents

Of the 1,103 professionals sampled, 23 were not eligible, five were not available after two attempts at the time of sampling, eight were interrupted during the course of the interview, and 46 refused to participate. Consequently, 1,021 professionals participated in the study (93% response rate). Although we did not gather information on nonrespondents, most of the 46 nonrespondents who refused to participate cited lack of time as the reason they were unable to participate (36), with other nonrespondents citing other obligations (four), fear of reprisal (one), and opposition to the study (three).

#### Sociodemographic characteristics

Professionals were predominantly female (67%) with a mean age of 36 y. Fifty-six percent were nurses, 31% were physicians, and 12% were certified midwives (Table 2).

**Table 2:**
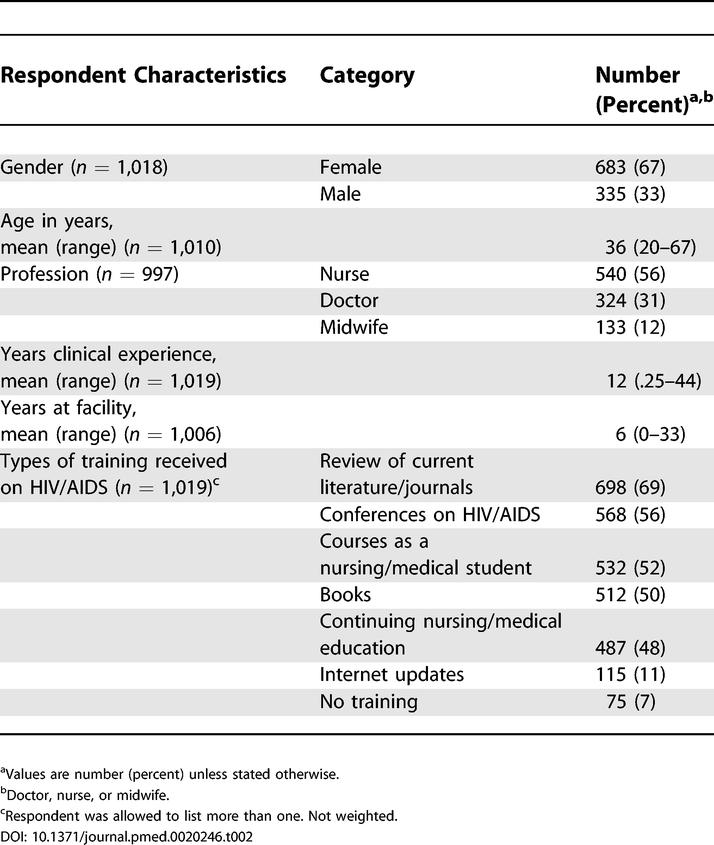
Demographic Characteristics among 1,021 Respondents

#### HIV/AIDS training

Most professionals reported having some training on HIV/AIDS (Table 2). Current literature (69%), conferences (56%), and courses as a student (52%) were most frequently reported by professionals as the sources of this training. Seven percent reported having no training on HIV/AIDS at all.

### Testing and Consent

#### Practices

Seventeen percent of surveyed health-care professionals reported that their facility had a written HIV testing policy (Table 3). Respondents indicated that the policies included requirements for informed consent (58%), pre-test counseling (53%), post-test counseling (52%), and post-test referral (29%).

**Table 3:**
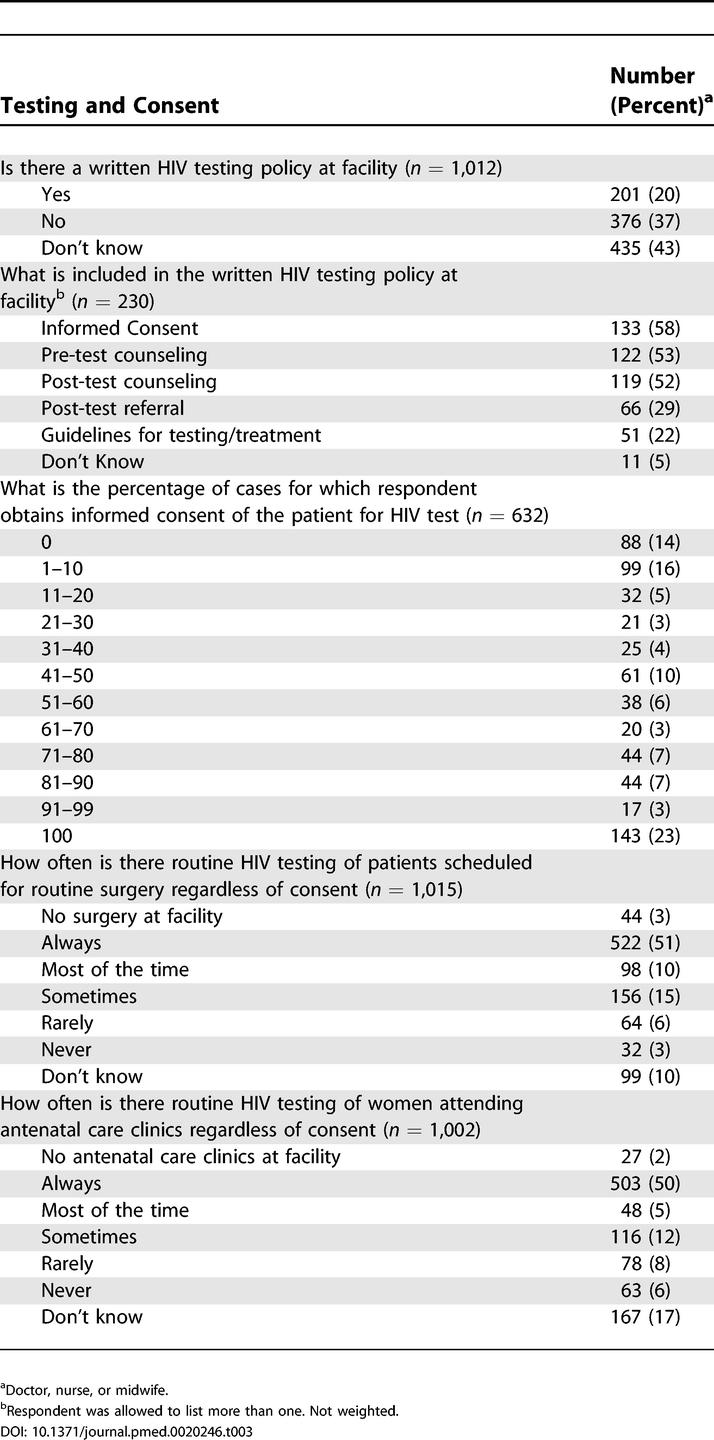
HIV/AIDS Testing, and Consent, Practices

Over 50% of professionals reported obtaining informed consent of patients for HIV tests half of the time or less, including 14% who reported never obtaining consent for HIV tests (Table 3). Fifty-four percent of respondents reported that, regardless of consent, routine HIV testing of all patients scheduled for surgery always took place at their facilities, and 50% reported such routine HIV testing of all women attending antenatal care clinics. Providers who reported that they lacked adequate training in HIV/AIDS treatment and ethics had 50% higher odds of reporting that they failed to obtain informed consent for HIV tests (more than 50% of the time) compared to providers reporting adequate training in these areas (odds ratio (OR) 1.53, 95% confidence interval [CI] 1.17–2.01).

#### Attitudes

Ninety-one percent of professionals agreed that staff and health-care professionals should be informed when a patient is HIV-positive so they can protect themselves (Table 4). Over three-quarters of respondents (78%) agreed that there are circumstances when it is appropriate to test a patient without his or her knowledge or permission. Fifty-seven percent of participants believed that relatives and sexual partners of patients with HIV/AIDS should be notified of the patient's status even without the patient's consent. Forty-six percent of professionals thought that the charts or beds of patients with HIV should be marked so that health facility workers know the patient's status.

**Table 4:**
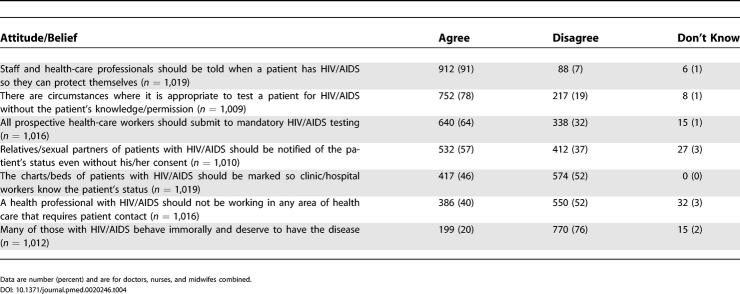
Provider Attitudes and Beliefs about HIV Testing, Consent, and Disclosure

Forty percent believed that health-care professionals with HIV/AIDS should not be working in any area of the health professions that requires patient contact. Twenty percent of respondents agreed that many of those who have HIV/AIDS behaved immorally and deserve the disease (Table 4).

Providers working in facilities that did not always practice universal precautions (65% citing lack of sufficient materials as the reason) were significantly more likely than those working in facilities that always observed universal precautions to agree that people with HIV/AIDS should not be employed in the health field (OR 1.43, 95% CI 1.09–1.74) and should not work in areas that require patient contact. They also had higher odds of agreeing that under certain circumstances, patients could be tested for HIV without their knowledge or permission (OR 1.63, 95% CI 1.14–2.33) Working in a facility that did not always practice universal precautions, being a nurse or midwife, and reporting inadequate training in HIV/AIDS treatment were all associated with agreeing that patients with HIV/AIDS should be on a separate ward in a hospital or clinic. Nurses and midwives both had more than five times the odds of agreeing that people with HIV/AIDS should not be employed in the health field than doctors and that the charts or beds of HIV patients should be marked. Nurses and midwives also had almost twice the odds of physicians of agreeing that under certain circumstances it is acceptable to test patients for HIV without their consent or knowledge.

### Treatment and Care

#### Practices

Among health-care professionals, the three most important concerns about treating patients with HIV/AIDS were fear of becoming contaminated (81%), contamination of facility, materials, or instruments (17%), and not having materials needed to treat them (10%) (Table 5). Seventy-two percent of respondents reported that universal precautions were always practiced in the facilities in which they worked. Lack of materials—reported by 65% of professionals—was cited as the main reason for non-practice of universal precautions (Table 5).

**Table 5:**
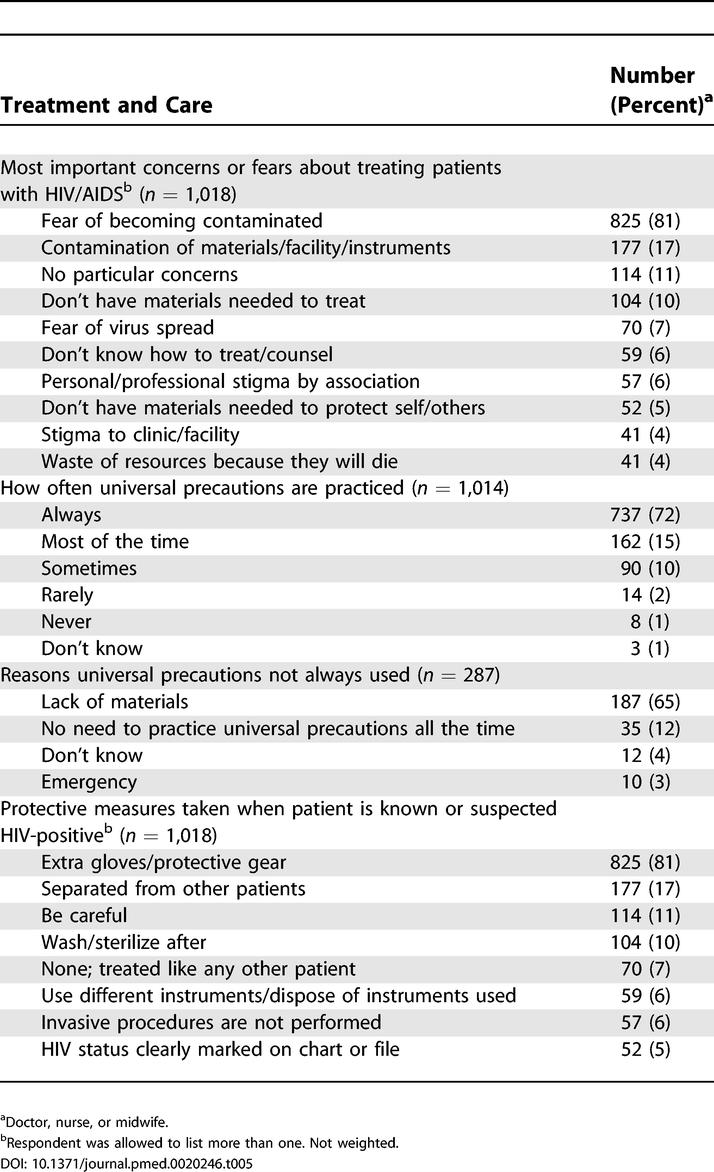
HIV/AIDS Treatment and Care Practices

Nine percent of professionals reported refusing to care for a patient with HIV/AIDS, and 9% indicated that they had refused a patient with HIV/AIDS admission to a hospital (Table 6). Sixty-six percent had observed other health-care professionals refusing to care for a patient with HIV/AIDS, and 43% had observed others refusing a patient with HIV/AIDS admission to a hospital. While less than one percent of professionals reported verbally mistreating a patient with HIV/AIDS, 27% of respondents reported seeing others verbally mistreat patients with HIV/AIDS.

**Table 6:**
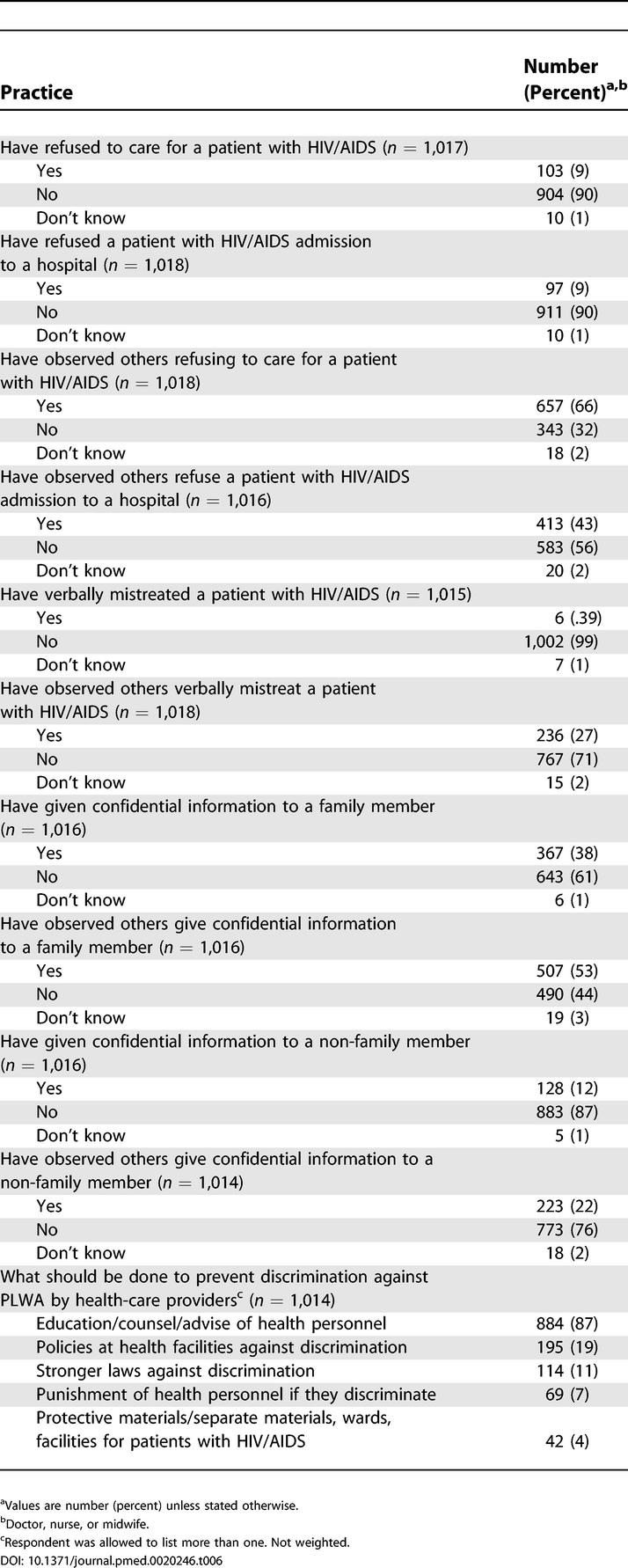
Assessment of Practices toward Patients with HIV/AIDS

Thirty-eight percent of professionals reported giving confidential information to a patient's family member without the patient's consent, and 53% had observed this behavior. Twelve percent of professionals reported giving confidential information to a person not related to a patient without consent, and 22% had observed this behavior (Table 6).

Providers who reported inadequate training in HIV/AIDS treatment and in ethics were significantly more likely to have refused to treat a patient with HIV/AIDS than those reporting adequate training in those two areas (OR 2.06, 95% CI 1.31–3.22). Providers working in facilities that did not always practice universal precautions were not more likely to have refused care to a patient themselves but were significantly more likely to report having observed other providers refuse to care for a patient with HIV/AIDS (OR 1.09, 95% CI 1.01–1.45). There were no differences among specialties in reporting having refused to care for a patient with HIV/AIDS.

#### Attitudes

To prevent discrimination by health-care professionals against patients with HIV/AIDS, most participants (87%) indicated that health-care professionals who engage in discriminatory practices should be educated and counseled. Health facility policies against discrimination were cited as solutions by 19% of professionals, and stronger laws against discrimination were suggested by 11% (Table 6).

Ninety-four percent indicated that medications to treat opportunistic infections may prolong the life of a patient who is HIV-positive (Table 7). Over half (59%) of professionals agreed that people with HIV/AIDS should be on a separate ward in a hospital or clinic. Forty-eight percent of participants expressed their belief that a person with HIV/AIDS cannot be treated effectively in their facility. Forty percent of health-care professionals reported that it is possible to determine a person's HIV status by looking at him or her, and 21% agreed that they could refuse to treat a patient with HIV/AIDS to protect themselves and their family. Twelve percent expressed agreement with the statement that treatment of opportunistic infections in patients with HIV/AIDS wastes resources, and 8% agreed that treating someone with HIV/AIDS is a waste of precious resources.

**Table 7:**
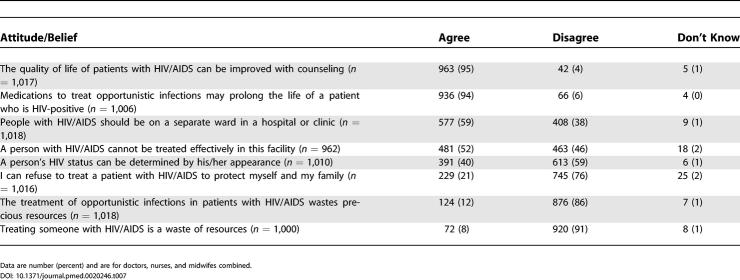
Provider Attitudes and Beliefs regarding Treatment and Care of Patients with HIV/AIDS

Nurses had higher odds than physicians of agreeing that treating opportunistic infections in patients with HIV/AIDS is a waste of resources (OR 2.14, 95% CI 1.35–3.40), but physicians were 50% more likely than nurses to agree that they could refuse to treat a patient with HIV/AIDS to protect themselves and their family. Respondents who reported inadequate training in HIV/AIDS treatment also were significantly more likely to agree it was acceptable to refuse to treat a patient for these reasons (OR 1.34, 95% CI 1.31–3.22). Physicians had significantly higher odds than either nurses or midwives of agreeing that there were circumstances under which it was appropriate to reveal a person's HIV status to others without the patient's knowledge or permission.

## Discussion

Most health-care professionals in the four states where the study was conducted appeared to be providing care to patients who were HIV-positive and complying with their ethical responsibilities despite their lack of training on HIV/AIDS and their having insufficient supplies of materials needed for treatment and prevention in the facilities where they work. A significant number, however, reported engaging in discriminatory and/or unethical behavior. These practices are corrosive to the health professions as they taint all health professionals and erode trust in them. They also represent missed opportunities for prevention, positive living education, and treatment, thereby undermining Nigeria's concerted national efforts to address the HIV/AIDS epidemic. Our study findings suggest that there are several factors that may contribute to such discriminatory and/or unethical behavior by health-care professionals against people with HIV/AIDS in Nigeria.

The vast majority of professionals expressed an interest in additional information and suggested education as a way to address discriminatory behaviors by their colleagues. An immediate investment to ensure the education of all existing clinical staff about HIV/AIDS, including modes of transmission, universal precautions, and the rights of PLWA would likely reduce the number of discriminatory practices towards PLWA and may improve these patients' care and access to health services. This assertion is supported by previous studies that demonstrate the effect of HIV/AIDS education of nurses and other health workers on their attitudes and behavior towards patients who are HIV-positive in Nigeria and elsewhere [24–26]. These studies also suggest that education about scientific matters is not likely to be sufficient to achieve change in practice and that educational programs may also need to address attitudes and cultural beliefs.

This study further suggests that the lack of protective and other materials needed to treat and prevent the spread of HIV and related conditions contributes to discriminatory behavior. While the issue of access to affordable antiretroviral treatment is the subject of much debate in Nigeria [13,27], many of the facilities in this study did not even have sufficient stocks of basic antibiotics to treat opportunistic infections. The lack of protective materials, documented in the health facility survey and cited also by professionals as the main reason for not applying universal precautions, contributes to discriminatory behavior in two ways. First, professionals lacking adequate protection may come to fear PLWA and fear may lead to discrimination [28–30]. Second, lack of resources also results in differential treatment practices that may contribute to stigmatization of PLWA.

In order to do their jobs safely and effectively, health professionals must be provided with adequate supplies of essential protective materials. Further, the lack of basic medications hampers the ability of health professionals to provide appropriate treatment. Without these materials, it is unlikely that education of health professionals and implementation of anti-discrimination policies alone will have the desired impact on practice.

It is likely that in other low-resource contexts, the absence of medications needed to treat HIV/AIDS-related illnesses, a lack of materials needed for protection of health personnel, and insufficient knowledge of health personnel about HIV/AIDS may contribute to discriminatory behavior towards people with HIV/AIDS. The role of these factors should be investigated. While addressing these factors may not eliminate all discriminatory behavior, these basic investments in the health-care sector are likely to result in improvements.

HIV infection is both a product of and a factor contributing to human rights violations [12]. The documented marginalization of certain groups, and their increased risk for infection with HIV in Nigeria [1], must be considered in light of this study. Misconceptions must be taken into account when developing education and training programs for professionals and the public. Nigerian health professionals are members of their society, one in which stigma and moral judgment appear to be attached to HIV/AIDS [6]. Twenty percent of respondents agreed that many of those who have HIV/AIDS behaved immorally and deserve the disease. As such, it is likely that governmental and facility policies and monitoring to reduce discriminatory practices in the health-care sector will be an important aspect of addressing these practices.

Numerous international and regional human rights instruments, to which Nigeria is a party [31], protect the rights of PLWA. These include [32–37] the African Charter on Human and People's rights [36], the Convention on Elimination of All Forms of Discrimination against Women [34], the Convention on the Rights of the Child [35], the International Convention on Elimination of All Forms of Racial Discrimination [37], the International Covenant on Economic, Social, and Cultural Rights [32], and the International Covenant on Civil and Political Rights [33]. Of these, only the African Charter and the Convention on the Rights of the Child have been incorporated into the domestic law of Nigeria [4,38]. Nigeria is also a signatory to the Universal Declaration of Human Rights [39].

The above instruments set out Nigeria's obligations to protect the rights of PLWA including the right to life [33], the right to education [36], the right to marry and found a family [33], the right to nondiscrimination [36], the right to share in the benefits of scientific advancements [32], the right to privacy [33], and the right to freedom of association [36].

Several of the instruments to which Nigeria is a party include the right to health [34–37,40]. The right to health, was first elaborated in the International Covenant on Economic, Social, and Cultural Rights, Article 12 [32], which states:

1. The States Parties to the present Covenant recognize the right of everyone to the enjoyment of the highest attainable standard of physical and mental health.
2. The steps to be taken by the States Parties to the present Covenant to achieve the full realization of this right shall include those necessary for:

(a) The provision for the reduction of the stillbirth-rate and of infant mortality and for the healthy development of the child;
(b) The improvement of all aspects of environmental and industrial hygiene;
(c) The prevention, treatment and control of epidemic, endemic, occupational and other diseases;
(d) The creation of conditions which would assure to all medical service and medical attention in the event of sickness.

In 2000, the Committee on Economic Social and Cultural Rights (ESCR Committee), responsible for interpretation and monitoring of the International Covenant on Economic, Social, and Cultural Rights, published General Comment 14 on the Right to the Highest Attainable Standard of Health [40]. The ESCR Committee determined that fulfillment of the right to health means that access to health services must not be limited based on discrimination on a prohibited ground, including HIV status .

In General Comment 14, the ESCR Committee also set out the core obligations of a state party to protect the right to health, which include ensuring “the right of access to health facilities, goods and services on a non-discriminatory basis, especially for vulnerable or marginalized groups,” the provision of essential drugs “as from time to time defined by WHO's Action Programme on Essential Drugs,” and ensuring “equitable distribution of all health facilities, goods and services.” In addition to these and other core obligations, the ESCR Committee also set out “obligations of comparable priority” , including a state party's obligation “to take measures to prevent, treat and control epidemic and endemic diseases,” “to provide education and access to information concerning the main health problems in the community, including methods of preventing and controlling them,” and “to provide appropriate training for health personnel, including education on health and human rights.”

The ESCR Committee also stated in General Comment 14 that “any person or group who is a victim of a violation of the right to health should have access to effective judicial or other appropriate remedies at both national and international levels.”As a state party, Nigeria is bound by the provisions of the International Covenant on Economic, Social, and Cultural Rights and the authoritative interpretations of the ESCR Committee. This study finds that some PLWA have been excluded from access to health care because of their HIV status and that, at this time, PLWA have no access to judicial or other remedial processes to address this. The data further suggest that inadequate education of health personnel about HIV/AIDS along with a lack of protective and treatment materials likely contribute to these behaviors by health professionals. It is therefore likely that Nigeria has not met its core obligations to fulfill and protect the right to health. The findings of this study suggest that, in order to fulfill its obligations, the government of Nigeria should continue to address gaps in policy and legislation and work together with the international community to ensure that health professionals receive the training, protective materials, and medications they need to treat PLWA.

International principles of medical ethics and Nigerian codes of conduct clearly provide for patient autonomy, i.e., the right to informed consent and confidentiality of patient information. In addition to representing violations of human rights, the denial of treatment and breaches of informed consent and confidentiality detailed in this paper contravene international principles of medical ethics and Nigerian health professional codes of conduct. The *Rules of Professional Conduct for Medical and Dental Practitioners in Nigeria* [41] states that “a doctor shall preserve absolute secrecy on all he knows about his patient even after the patient has died, because of the confidence entrusted to him.” The binding rules also state that “practitioners…must always obtain consent of the patient or the competent relatives...before embarking on any special treatment procedures with determinable risks.”

Nigerian medical practitioners also have a duty under these rules to report any unethical conduct by their peers to the Medical and Dental Council of Nigeria. According to the rules, “every doctor or dentist must be his brother's keeper, with regard to the observance and indeed the enforcement of the rules and regulations which guide the profession. Doctors and dentists should expose without fear or favour, before the Medical and Dental Council of Nigeria either directly or through the Nigerian Medical Association, any corrupt, dishonest, unprofessional or criminal act or omission on the part of any doctor or dentist.” There is no indication that this may have happened in the case of the breaches documented in this study. At the time of publication, no specific medical ethics principles on HIV and AIDS have been articulated by the Medical and Dental Council of Nigeria.

### Limitations

The study was conducted in four states in Nigeria with a total population of 17 million [2]. It is possible that these sites, though chosen at random from states with tertiary care facilities, may differ significantly from others in terms of resources and training provided to health-care providers. Although sampled systematically, it is possible that sampled facilities and health-care professionals may differ significantly from those that were not sampled in the four study states.

Although the findings of this study can not be generalized to Nigeria as a whole, it is likely that, depending on resources and training available to the health-care sector, the level of discriminatory behavior may differ in other parts of the country.

The apparent discrepancy between reported and observed behavior may indicate under- or overreporting of discriminatory behavior or may result from health-care professionals within the same institution having observed the same incidents.

While this study focused on HIV/AIDS, it is possible that health-care professionals also engage in inappropriate behavior toward or breach the confidentiality of people with other conditions. The health-care system in Nigeria is underfunded and suffers from fundamental problems including material scarcity and inadequacies in infrastructure, which may contribute to this behavior overall [4,42,43]. We did not specifically ask clinicians to compare their treatment of patients positive for HIV with that of other patients. Even if health-care professionals engage in breaches of confidentiality and other inappropriate behavior toward patients with other conditions, however, it is likely that the consequences of such actions may be worse for patients positive for HIV than for patients with other conditions.

Despite efforts to ensure privacy during interviews, the lack of privacy, or concern about job status, may have resulted in an underreporting of discriminatory behavior and/or an overreporting of “correct” practices or attitudes. Although interviewers were careful to explain that there would be no material gain or penalty to the respondent or his or her facility from participation in the study, the responses may have been inaccurate if respondents judged it in their material or political interest to exaggerate or conceal certain behaviors.

### Conclusion

Despite these limitations, the study documents a significant proportion of health professionals in four states in Nigeria as reporting discriminatory attitudes and engaging in discriminatory and unethical behavior toward patients with HIV or AIDS, including denial of care, breach of confidentiality, and non-consented HIV testing. The breaches of confidentiality and testing for HIV without informed consent reported by participants are in contravention of international principles of medical ethics [44], and are also breaches of the Nigerian physician code of conduct [41]. The study identifies four factors that may contribute to this behavior: lack of correct information and education about HIV/AIDS and prevention of infection, lack of protective materials needed for the practice of universal precautions, lack of materials needed to care for and treat patients with HIV/AIDS, and prevailing attitudes about PLWA. This study suggests that adequately addressing these discriminatory practices and attitudes requires targeted education of health professionals and provision of adequate resources to health-care facilities combined with instituting and enforcing anti-discrimination policies.

**Patient Summary**

**Background**

People living with HIV/AIDS experience discrimination all over the world, often in both their private and professional lives, and sometimes by health-care personnel, including doctors and nurses.

**Why Was This Study Done?**

The researchers, who are part of a not-for-profit organization called Physicians for Human Rights, wanted to find out whether discrimination existed among health workers in Nigeria. Nigeria is home to one in 11 people living with HIV/AIDS, and antiretroviral drugs to treat patients are not widely available in the country.

**What Did the Researchers Do?**

They developed a survey and trained interviewers to get answers from 1,021 Nigerian health-care professionals—including 324 physicians, 541 nurses, and 133 midwives—about their attitudes and actions towards patients with HIV/AIDS. These health-care professionals worked in 111 different facilities in four of Nigeria's 36 states. The survey included questions such as “have you refused to care for an HIV/AIDS patient?” and “do you believe that a person's HIV status can be determined by his/her appearance?”

**What Did They Find?**

They found that while most of the interviewed people said that they treated people with HIV/AIDS in accordance with ethical and medical guidelines, a significant number of them reported attitudes and behavior that the researchers found worrisome. For example, 20% agreed that many individuals with HIV/AIDS had behaved immorally and deserved their infection, and 8% felt that treating someone with HIV/AIDS was a waste of resources. It didn't seem that there were big differences between the three groups (doctors, nurses, and midwives). Negative attitudes were higher among people from facilities that were not always able to take precautions against HIV infection (owing to lack of supplies). The same was true for facilities that did not have antiretroviral drugs to treat patients.

**What Does This Mean?**

It means that quite a few of the professionals surveyed—whose job it is to care for patients with HIV/AIDS—have negative attitudes towards them, and some of them have behaved in discriminating ways.

**What Next?**

It is not clear how representative the attitudes and behaviors reported by the participants in this study are, and future studies in Nigeria and in other countries are necessary to answer that question. Despite its limitations, this study suggests three things might help to reduce discrimination: educating health-care workers about HIV/AIDS and ethics, making sure that all facilities take appropriate precautions against HIV transmission, and making sure that all facilities can provide adequate care for patients with HIV/AIDS, including antiretroviral drugs.

**Additional Online Resources**

Information about stigmatization of people living with AIDS can be found at the following sources.

Joint United Nations Program on HIV/AIDS:

http://www.unaids.org/en/in+focus/hiv_aids_human_rights/stigma_discrimination.asp

United Kingdom National AIDS Trust:

http://www.areyouhivprejudiced.org/AboutUs.aspx

An article from the *Indian Journal of Medical Ethics:*

http://www.issuesinmedicalethics.org/082mi060.html

Physicians for Human Rights home page: http://www.phrusa.org/

Physicians for Human Rights Web page on HIV and human rights:

http://www.phrusa.org/campaigns/aids/aidsandhr.html

WHO work on HIV and human rights:

http://www.who.int/hhr/activities/publications/en/index.html

## Abbreviations

CI: confidence interval
ESCR Committee: Committee on Economic Social and Cultural Rights
OR: odds ratio
PHR: Physicians for Human Rights
PLWA: people living with HIV/AIDS
